# Preoperative Weight Gain Is Not Related to Lower Postoperative Weight Loss, But to Lower Total Weight Loss up to 3 Years After Bariatric-Metabolic Surgery

**DOI:** 10.1007/s11695-023-06835-5

**Published:** 2023-11-03

**Authors:** Anne Jacobs, May Al Nawas, Laura N. Deden, Lea M. Dijksman, Evert-Jan G. Boerma, Ahmet Demirkiran, Eric J. Hazebroek, M. (René) J. Wiezer, Wouter J.M. Derksen, Valerie M. Monpellier

**Affiliations:** 1 Nederlandse Obesitas Kliniek, Amersfoortseweg 43, 3712 BA Huis ter Heide, Utrecht The Netherlands; 2 Department of Surgery, Leiden University Medical Center, Leiden, The Netherlands; 3 Department of Surgery, St. Antonius Hospital, Nieuwegein, The Netherlands; 4 Department of Surgery, Vitalys Clinic, Part of Rijnstate Hospital, Arnhem, The Netherlands; 5 Department of Research and Epidemiology, St. Antonius Hospital, Nieuwegein, The Netherlands; 6 Department of Surgery, Zuyderland Hospital, Heerlen, The Netherlands; 7 Department of Surgery, Rode Kruis Hospital, Beverwijk, The Netherlands

**Keywords:** Preoperative weight loss, Mandatory weight loss, Postoperative weight loss, Bariatric-metabolic surgery

## Abstract

**Introduction:**

Weight loss prior to bariatric-metabolic surgery (BMS) is recommended in most bariatric centers. However, there is limited high-quality evidence to support mandatory preoperative weight loss. In this study, we will evaluate whether weight gain prior to primary BMS is related to lower postoperative weight loss.

**Methods:**

A retrospective analysis of prospectively collected data was performed. Preoperative weight loss (weight loss from start of program to day of surgery), postoperative weight loss (weight loss from day of surgery to follow-up), and total weight loss (weight loss from start of program to follow-up) were calculated. Five groups were defined based on patients’ preoperative weight change: preoperative weight loss of >5 kg (group I), 3–5 kg (group II), 1–3 kg (group III), preoperative stable weight (group IV), and preoperative weight gain >1 kg (group V). Linear mixed models were used to compare the postoperative weight loss between group V and the other four groups (I–IV).

**Results:**

A total of 1928 patients were included. Mean age was 44 years, 78.6% were female, and preoperative BMI was 43.7 kg/m^2^. Analysis showed significantly higher postoperative weight loss in group V, compared to all other groups at 12, 24, and 36 months follow-up. Up to three years follow-up, highest total weight loss was observed in group I.

**Conclusion:**

Weight gain before surgery should not be a reason to withhold a bariatric-metabolic operation. However, patients with higher preoperative weight loss have higher total weight loss. Therefore, preoperative weight loss should be encouraged prior to bariatric surgery.

**Graphical Abstract:**

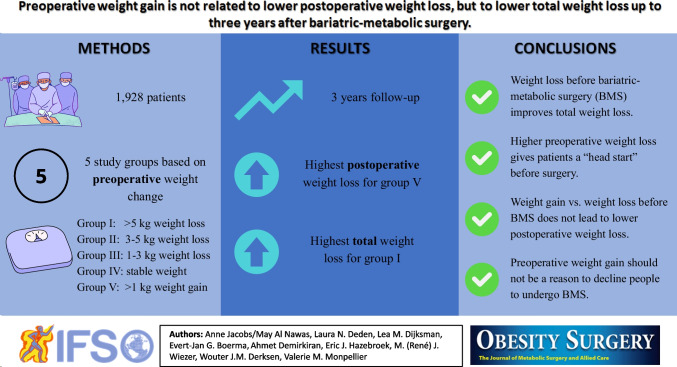

**Supplementary Information:**

The online version contains supplementary material available at 10.1007/s11695-023-06835-5.

## Introduction

Bariatric-metabolic surgery (BMS) is the most effective treatment for severe obesity. It results in significant weight loss and resolution of obesity-related medical problems [1, 2]. To improve post-bariatric outcomes, some advocate (mandatory) preoperative weight loss. Furthermore, many insurance companies require adherence to a preoperative weight loss program or a specific amount of weight loss as a prerequisite for approval for BMS [3, 4]. However, it is unclear whether preoperative weight loss is related to postoperative weight loss after BMS and if patients with weight gain prior to surgery have lower postoperative weight loss.

Preoperative weight loss has been hypothesized to be a marker to identify patients who are compliant to the treatment program [3]. Patients who achieve weight loss before surgery are believed to be more motivated and adapted to the new postoperative lifestyle and, thus, more successful in reaching and sustaining satisfactory postoperative weight loss [5–9].

Since the concept of preoperative weight loss was first introduced by the National Institutes of Health consensus panels in the 1990s, studies have reported conflicting and inconsistent results on the effects of preoperative weight loss on post-bariatric weight loss [8–13]. The most important reason for this is that the definition of preoperative weight loss is often unclear and differs between studies [14–19]. Preoperative weight loss is often included in the calculation of total postoperative weight loss, making it impossible to study the exact relationship between preoperative weight loss and postoperative weight loss [20–22]. Moreover, there is heterogeneity of the study designs, and often a relatively small number of patients are included [3, 8–13]. Lastly, most studies focus only on short term weight loss (12 months follow-up point) [6–8, 10]. Hence, there is limited high-quality evidence to support or refute mandatory preoperative weight loss for patients who will undergo BMS.

In this study, we analyze data of a large multicenter cohort with prospectively collected data up to 3 years after surgery. We aim to evaluate whether weight gain prior to primary BMS is related to lower postoperative weight loss.

## Methods

### Study Design

A retrospective analysis of prospectively collected data was performed to evaluate the effect of preoperative weight loss in patients undergoing elective BMS. This study was approved by the Medical Ethical Committee Zuyderland & Zuyd (METCZ20190097) and the Local Ethics Committees in the participating bariatric centers. The study was reported in accordance with the Strengthening the Reporting of Observational Studies in Epidemiology (STROBE) reporting guideline for cohort studies [23].

### Setting

The cohort consisted of patients who underwent primary BMS between January 1st and December 31st of 2017 in one of the following hospitals, all located in the Netherlands: St. Antonius hospital Nieuwegein, Rijnstate hospital Arnhem, Rode Kruis hospital Beverwijk, and Zuyderland Medical Center Heerlen. All patients followed a pre- and postoperative interdisciplinary program at the Nederlandse Obesitas Kliniek (Dutch Obesity Clinic, NOK). The NOK is the largest outpatient clinic center in the Netherlands for the treatment of obesity. At the NOK care centers, patients follow an interdisciplinary treatment program [24]. The preoperative program consists of six group sessions spread over 6 weeks, where all patients were advised to aim for a weight loss of 3–5 kg. The postoperative program included a comprehensive 1-year lifestyle change program. Postoperative follow-up visits are attended yearly until 5 years after surgery.

### Patient Population

All patients were screened according to International Federation for the Surgery of Obesity criteria [25] and underwent one of the following primary laparoscopic BMS procedures: Roux-en-Y gastric bypass (RYGB), sleeve gastrectomy (SG), or banded Roux-en-Y gastric bypass (bRYGB). Patients with a medical history of BMS and patients who took part in an individual treatment program (e.g., because of linguistic barriers or psychopathology) were excluded. Cases missing weight data at the start of the preoperative care program or at the day of surgery were also excluded.

### Data Source

Patients were included from the database of the NOK. Data was collected from the NOK patient files, the hospital patient files, and was linked to the Dutch national registry Dutch Audit of Treatment of Obesity (DATO). The DATO is a mandatory registry containing patients’ data from all hospitals performing BMS in the Netherlands since January 2015 [26].

### Variables

Data was collected until January 2021. Study data collected from the NOK database include patient demographics (age and gender), surgical procedure, preoperative obesity-associated medical problems (diabetes mellitus, hyperlipidemia, hypertension, and obstructive sleep apnea), and the weight measured at the clinic at the start of the preoperative care program as well as at follow-up appointments up to 3 years after surgery (3, 6, 12, 18, 24, and 36 months).

The preoperative American Society of Anesthesiologists (ASA) physical status classification, weight measured in the hospital at the day of surgery, perioperative complications, and severe short-term <30-day postoperative complications according to the Clavien-Dindo Classification of Surgical Complications grade ≥III [27] were collected from the hospital patient files and DATO database.

### Preoperative Weight Change

Preoperative weight change (PWC) was defined as the difference in kilograms between the weight at the start of the preoperative care program (first group session) and the weight measured at the day of the bariatric procedure:

$$\mathrm{PWC}={\mathrm{weight}}_{\mathrm{day}\;\mathrm{of}\;\mathrm{surgery}}-{\mathrm{weight}}_{\mathrm{start}\;\mathrm{preoperative}\;\mathrm{care}\;\mathrm{program}}$$

Based on their PWC, patients were stratified into five groups: those who lost >5 kg (group I), those who lost 3–5 kg (group II), those who lost 1–3 kg (group III), those who had a stable weight with a range of 1 kg (group IV), and those who gained more than 1 kg (group V). These cut-offs were believed to be clinically relevant, since patients are advised to not gain weight and to lose 3–5 kg. This results in five groups with an equal range of 2 kg between the groups.

### Outcome Measures

**Fig. 1:**
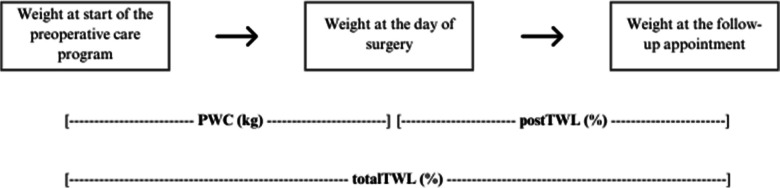
Overview of the used definitions of weight loss

Weight loss was calculated as percentage total weight loss (%TWL) and absolute change in body mass index (ΔBMI). Figure 1 provides an overview of the used definitions of weight loss.

Postoperative TWL was defined as the percentage of weight difference between the weight at the day of surgery and follow-up weight:

$$\mathrm{postTWL}=\left[\left.\left({\mathrm{weight}}_{\mathrm{day}\;\mathrm{of}\;\mathrm{surgery}}-{\mathrm{weight}}_{\mathrm{follow}\;\mathrm{up}}\right)/{\mathrm{weight}}_{\mathrm{day}\;\mathrm{of}\;\mathrm{surgery}}\right\}\right]\times100\%$$

Total TWL was defined as the percentage of weight difference between weight at the start of the preoperative care program and follow-up weight:

$$\mathrm{totalTWL}=\left[\left.\left({\mathrm{weight}}_{\mathrm{start}\;\mathrm{care}\;\mathrm{program}}-{\mathrm{weight}}_{\mathrm{follow}\;\mathrm{up}}\right)/{\mathrm{weight}}_{\mathrm{start}\;\mathrm{care}\;\mathrm{program}}\right\}\right]\times100\%$$

Total ΔBMI was defined as the difference in BMI points between the BMI at the start of the preoperative care program and follow-up BMI:

$$\Delta\mathrm{BMI}={\mathrm{BMI}}_{\mathrm{follow}\;\mathrm{up}}-{\mathrm{BMI}}_{\mathrm{start}\;\mathrm{preoperative}\;\mathrm{care}\;\mathrm{program}}$$

### Statistical Analysis

Descriptive statistics were used to summarize baseline characteristics. Categorical data were expressed as number (percentages). Continuous data were expressed as mean ± standard deviation when normally distributed, otherwise as median [interquartile range]. Baseline differences between the PWC groups were evaluated by using analysis of variance (ANOVA) for continuous variables and chi-square tests for categorical variables.

Linear mixed model analysis was used to compare the change in postTWL over time in the five groups. The groups were included as covariates in the fixed part, where group V (> 1 kg weight gained) was used as reference group. After determining the best-fitting model with random intercept and/or slope, only a random intercept for subject (patient) was added in the crude model (model 1). Age at baseline, sex, BMI at start of the preoperative care program, and (natural logarithm of) time between start preoperative care program and day of surgery were considered as important confounders and were included as covariates in model 2. Model 3 included the following additional confounders: preoperative ASA score, number of associated medical problems at baseline, type of BMS, and the bariatric center where the patients were treated. The results of the models for 12, 24, and 36 months postTWL were reported as (adjusted) regression coefficients (*β*) with 95% confidence interval (95% confidence intervals, CI) and the *p*-value.

Statistical analysis was performed using SPSS (IBM Corp. Released 2019. IBM SPSS Statistics for Windows, Version 26.0. Armonk, NY: IBM Corp). Weight changes were presented in graphs, created by using the package “ggplot2” in R (R Foundation for Statisical Computing, Vienna, Austria. URL https://www.R-project.org). All statistical tests were two-tailed, and *p*<.05 was considered statistically significant.

## Results

### Study Population

A total of 1945 patients underwent primary BMS in one of the participating bariatric centers in 2017 and followed a perioperative care program at the NOK. Seventeen patients (0.9%) were excluded due to missing weight data at the start of the treatment program and/or the day of surgery. A total of 1928 patients were included.

### Baseline Characteristics

The majority of patients was female (78.6%) and mean age was 44.1±11.4 years (Table 1). Median time interval between the start of the preoperative care program and BMS was 7.3 weeks [6.1–9.1]. The most frequently performed BMS was RYGB (*n*=1229, 63.7%), followed by SG (*n*=446, 23.1%), and bRYGB (*n*=253, 13.1%). A total of 34 (1.7%) patients had a perioperative (*n*=10, 0.5%) or severe short-term (*n*=24, 1.2%) complication within 30 days after surgery. There was no mortality in this cohort.

**Table 1:** Demographic characteristics of the total population (*n*=1928) presented as mean ± standard deviation or number (percentage)

*Variable*	*Value*
Age, years	44.1 ± 11.4
Female sex	1517 (78.6)
Bariatric surgery type	
Roux-en-Y gastric bypass	1229 (63.7)
Sleeve gastrectomy	446 (23.1)
Banded Roux-en-Y gastric bypass	253 (13.1)
ASA score	
2	1040 (53.9)
3	866 (44.9)
4	15 (0.8)
Associated medical problems	
Hypertension	663 (34.4)
Type 2 diabetes	394 (20.4)
Dyslipidemia	383 (19.9)
Obstructive sleep apnea syndrome	338 (17.5)
BMI, kg/m^2^	
Start of preoperative care program	43.7 (±5.6)
Day of surgery	43.1 (±5.6)

*ASA* American Society of Anesthesiologists physical status classification, *BMI* body mass index

### Comparison Between Groups

In total, 296 patients were included in group I, 263 in group II, 447 in group III, 623 in group IV, and 299 in group V. An overview of all characteristics is shown in Table 2. The groups were comparable for most characteristics, though some differences were observed. Age was lower in group V compared to all other groups (*p*=.02). In group I and II, the percentage of people undergoing a banded RYGB was higher compared to the other groups (*p*<0.01). The frequency of type II diabetes was higher in group II and IV as compared to the other groups (*p*<0.01).

**Table 2:** Comparison of the groups according to preoperative weight change before bariatric surgery presented as mean ± standard deviation, median interquartile range, or number (percentage)

	Group I Weight loss (>5 kg)	Group II Weight loss (3–5 kg)	Group III Weight loss (1–3 kg)	Group IV Stable weight (−1 to +1 kg)	Group V Weight gain (>1 kg)	*p*-value
Number of patients	296	263	447	623	299	
Age, years	44.8 ± 10.4	45.0 ± 11.4	44.9 ± 11.1	43.6 ± 11.8	42.6 ± 12.0	0.02
Female	222 (75.0)	205 (77.9)	370 (82.8)	496 (79.6)	224 (74.9)	0.04*
Bariatric surgery type						<0.01*
Roux-en-Y gastric bypass	141 (47.6)	158 (60.1)	314 (70.2)	437 (70.1)	179 (59.9)	
Sleeve gastrectomy	38 (12.8)	59 (17.5)	96 (21.5)	152 (24.4)	101 (33.8)	
Banded Roux-en-Y gastric bypass	117 (39.5)	46 (22.4)	37 (8.3)	34 (5.5)	19 (6.4)	
ASA score						<0.01*
2	142 (48.0)	110 (41.8)	250 (55.9)	373 (55.9)	165 (55.2)	
3	147 (49.7)	151 (47.4)	193 (43.2)	242 (38.8)	133 (44.5)	
4	7 (2.7)	1 (0.4)	2 (0.4)	4 (0.6)	1 (0.3)	
Associated medical problems						
Hypertension	99 (33.4)	96 (36.5)	154 (34.5)	215 (34.5)	99 (33.1)	0.93
Type 2 diabetes	46 (15.5)	60 (22.8)	95 (21.3)	143 (23.0)	50 (16.7)	0.03*
Dyslipidemia	60 (20.3)	56 (21.3)	93 (20.8)	116 (18.6)	58 (19.4)	0.87
OSAS	58 (19.6)	52 (19.8)	80 (17.9)	96 (15.4)	52 (17.4)	0.44
BMI, kg/m^2^						
Start preoperative care program	44.6 ± 6.8	43.9 ± 5.5	43.2 ± 5.1	43.5 ± 5.2	44.0 ± 5.6	0.01*
Day of surgery	42.0 ± 6.6	42.5 ± 5.5	42.5 ± 5.1	43.5 ± 5.2	45.0 ± 5.7	<0.01*
Preoperative weight change						
Weight change before surgery, kg	−6.8 [−5.9 to −8.5]	-4.0 ± 0.6	−2.0 ± 0.6	0.0 ± 0.6	2.2 [1.5–3.7]	<0.01*
Weight change before surgery, %TWL	−5.8 [−5.0 to −7.2]	−3.3 ± 0.7	−1.6 ± 0.5	0.0 ± 0.5	1.7 [1.2–2.8]	<0.01*

*ASA* American Society of Anesthesiologists physical status classification, *OSAS* obstructive sleep apnea syndrome, *BMI* body mass index, *TWL* total weight loss. *Significant differences between groups

### Weight and Preoperative Weight Change

The mean BMI at the start of the preoperative care program was 43.7±5.6 kg/m^2^ and BMI at the day of surgery was 43.1±5.6 kg/m^2^ (Table 1). In group I, BMI at the start of the preoperative care program was higher as compared to group III and group IV (44.6±6.8 versus 43.2±5.1 and 43.5±5.2 kg/m^2^, respectively (Table 2, *p*=.01).

In the total population, mean preoperative weight change was −1.7±3.4 kg. In group I, median weight loss was −6.8kg [−5.9 to −8.5], and group V gained median +2.2 kg [1.5 to 3.7] between the start of the preoperative care program and the day of surgery.

### Total Weight Loss

**Fig. 2:**
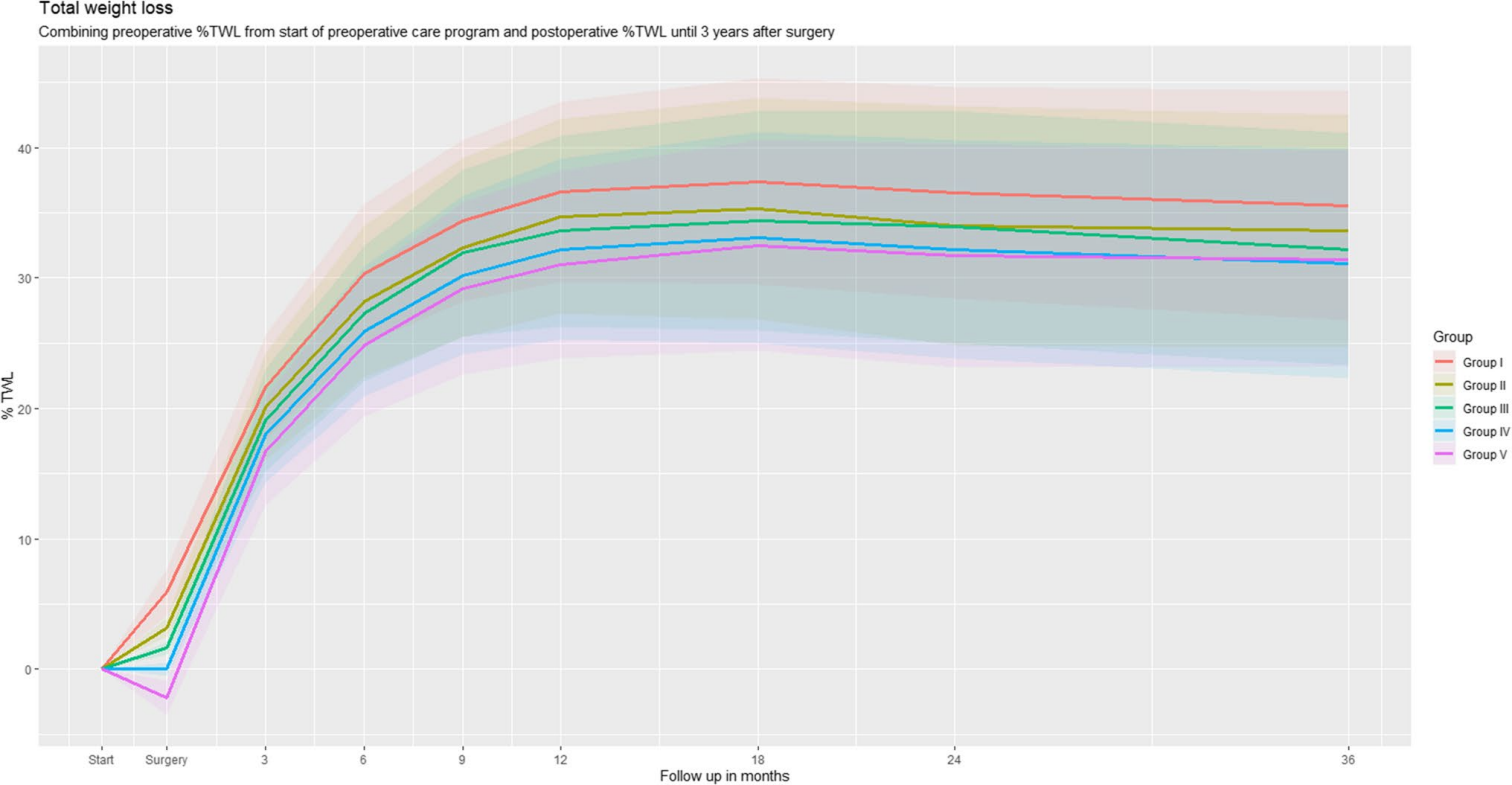
Preoperative weight change and postoperative total weight loss over time for each of the study groups

The weight change of the total bariatric program from the start of the preoperative care program until 3 years after BMS is presented in Figs. 2 (%TWL) and 3 (BMI) and Supplementary Table 1. The weight loss pattern was similar in all five groups, with a largest weight loss achieved at 18-month follow-up. At 3-year follow-up, mean totalTWL was 33.7±8.2 %, 32.6±8.6%, 31.7±8.7%, 31.1±8.8 %, and 32.1±8.5 % for the groups I, II, III, IV, and V, respectively. Mean ΔBMI was also highest in group I, −15.1±4.9 compared to −14.3±4.6, −13.8±4.4, −13.4±4.2, and −14.3±4.4 for, respectively, group II, III, IV, and V. Loss to follow-up was 30.5% at 3-year follow-up (Supplementary Table 1).

**Fig. 3:**
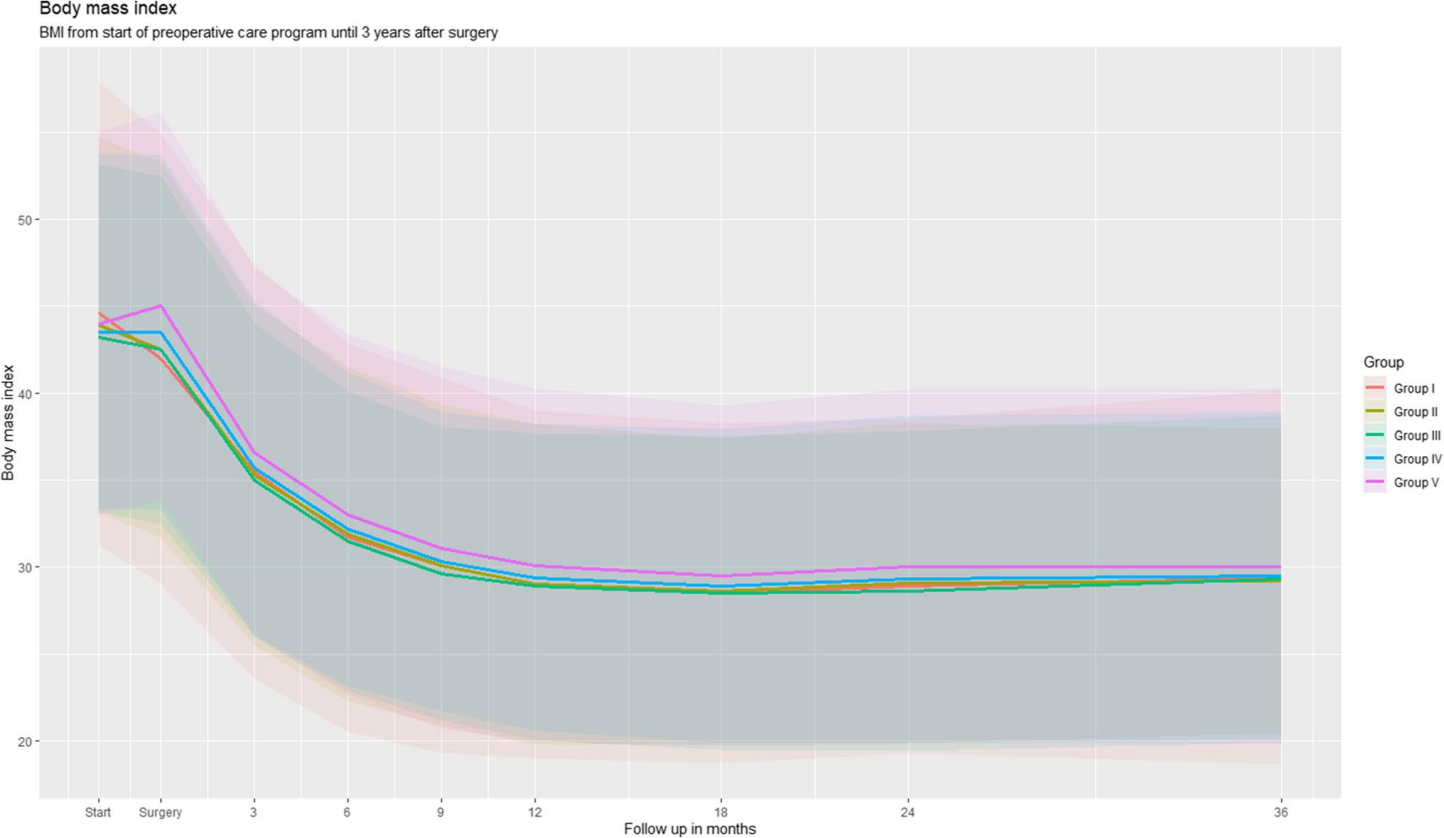
Body mass index over time for each of the study groups

### Postoperative Weight Loss

Highest postTWL at 3-year follow-up was observed in group V (33.5±8.3%) compared to group I (29.6±8.8), group II (30.4±9.0), group III (30.6±8.9), and group IV 31.1±8.8 (Fig. 2 and Supplementary Table 1). Adjusted for potential confounders, group V had a significant higher postTWL at 12-, 24-, and 36-month follow-up, compared to all other groups (*p*<0.01, Table 3). For example, adjusted for all potential confounders, BMS lead to a 4.03% lower postTWL in group I (lost >5 kg) compared to group V (gain > 1 kg) (*β* −4.03; 95% CI −5.21 to −2.85, *p*<0.01) (Table 3).

**Table 3:** Mixed models analysis for the percentage postoperative total weight loss (postTWL), group V (weight gain >1 kg) is the reference group

	12 months			24 months			36 months		
	*β*	*95% CI*	*p-value*	*β*	*95% CI*	*P-value*	*β*	*95% CI*	*p-value*
Group I Weight loss >5 kg									
Model 1^a^	−2.42	−3.55 to −1.30	<0.01	−3.05	−4.20 to −1.89	<0.01	−3.71	−4.91 to −2.52	<0.01
Model 2^b^	−2.76	−3.86 to −1.67	<0.01	−3.39	−4.51 to −2.27	<0.01	−4.07	−5.23 to −2.91	<0.01
Model 3^c^	−2.76	−3.87 to −1.64	<0.01	−3.36	−4.50 to −2.22	<0.01	−4.03	−5.21 to −2.85	<0.01
Group II Weight loss 3–5 kg									
Model 1^a^	−1.68	−2.84 to −0.52	0.01	−2.92	−4.12 to −1.71	<0.01	−2.43	−3.67 to −1.19	<0.01
Model 2^b^	−1.96	−3.01 to −0.84	<0.01	−3.20	−4.37 to −2.03	<0.01	−2.72	−3.93 to −1.52	<0.01
Model 3^c^	−1.60	−2.70 to −0.49	0.01	−2.83	−3.99 to −1.68	<0.01	−2.35	−3.54 to −1.16	<0.01
Group III Weight loss 1–3 kg									
Model 1^a^	−1.07	−2.10 to −0.05	0.04	−1.44	−2.50 to −0.39	0.01	−2.09	−3.18 to −0.99	<0.01
Model 2^b^	−1.26	−2.25 to −0.26	0.01	−1.62	−2.65 to −0.60	<0.01	−2.27	−3.34 to −1.21	<0.01
Model 3^c^	−1.33	−2.29 to −0.37	0.01	−1.69	−2.68 to −0.69	<0.01	−2.32	−3.35 to −1.29	<0.01
Group IV Stable weight									
Model 1^a^	−0.94	−1.90–0.03	0.06	−1.69	−2.69 to −0.69	<0.01	−1.97	−3.01 to −0.94	<0.01
Model 2^b^	−0.92	−1.85–0.01	0.05	−1.66	−2.63 to −0.70	<0.01	−1.96	−2.96 to −0.96	<0.01
Model 3^c^	−1.00	−1.90 to −0.09	0.03	−1.73	−2.67 to −0.80	<0.01	−2.02	−3.00 to −1.05	<0.01

β = coefficient of regression, e.g., difference in % postTWL between group V (reference group) and the other groups; *95% CI* 95% confidence interval

^a^Crude model. ^b^Model adjusted for age, sex, preoperative BMI, time between start preoperative program and day of surgery. ^c^Model adjusted for covariates in model 2 plus hospital, ASA score, comorbidities (0, 1, 2, 3, or 4 conditions), and type of bariatric surgery (RYGB, GS, bRYGB)

## Discussion

In this large retrospective cohort study, the goal was to study if patients with weight gain prior to primary BMS had lower postoperative weight loss. Our data show that the highest postoperative weight loss was observed in the group with preoperative weight gain, with a difference at 36 months of 4.03% when compared to the group that had the highest preoperative weight loss. Therefore, preoperative weight gain should not be used as a negative “indicator” for postoperative weight loss and patients should not be denied BMS, solely based on their preoperative weight change. Total weight loss (combination of pre- and postoperative) was highest in the group of patients with the highest preoperative weight loss (maximum difference 2.5%). The higher total weight loss seems to be entirely attributed to the weight lost before surgery.

Previous (systematic) reviews and meta-analyses addressing this issue also concluded there was no evidence that weight loss prior to surgery improved weight loss [9, 10, 12, 13, 28]. Thus, the assumption that more preoperative weight loss indicates a greater level of motivation and leads to better weight loss after surgery appears to be unfounded. Our finding that higher total weight loss was observed in patients with the highest preoperative weight loss suggests that preoperative weight loss may give patients a “head start” in their weight loss journey. Therefore, preoperative weight loss should still be encouraged to all patients applying for BMS.

A key implication is that clear and accurate definitions of outcome measurements for weight loss are essential. In the current study, there were strict definitions of preoperative weight loss, postoperative weight loss, and total weight loss. Often, there are no clear definitions of preoperative and postoperative weight loss; the studies might have investigated total weight loss, instead of postoperative weight loss [20–22].

The strength of the current study is its strict and specific definitions of preoperative weight loss, postoperative weight loss, and total weight loss and the large multicentre patient group with high follow-up rates at 36 months after surgery. A limitation of the current study is that the main outcomes were limited to weight loss. Preoperative weight loss may also affect other BMS outcomes, such as the risk of perioperative complications. Finally, due to the retrospective design, the current study does not provide information on which treatments or patient characteristics are associated with preoperative weight change.

## Conclusion

Preoperative weight gain is not related to lower postoperative weight loss up to 3 years after BMS and should not be a reason to deny patients access to treatment. However, patients who lose weight before surgery have higher total weight loss, because of the weight lost before the procedure. Therefore, preoperative weight loss should be encouraged prior to bariatric surgery.

### Supplementary Information

ESM 1 (DOCX 14 kb)
