# Supplementary material for: Preoperative Weight Gain Is Not Related to Lower Postoperative Weight Loss, But to Lower Total Weight Loss up to 3 Years After Bariatric-Metabolic Surgery
Source: Obes Surg. 2023 Nov 3;33(12):3746–54. doi: 10.1007/s11695-023-06835-5 (PMC10687109; doi:10.1007/s11695-023-06835-5)
Supplement: Supplementary file 1 — (DOCX 14 kb) [file 11695_2023_6835_MOESM1_ESM.docx]

| Supplementary table 1  Overview preoperative weight change, postoperative weight and total weight loss up to 36 months after surgery for the groups, presented as mean ± standard deviation, and overview of missing data per follow-up moment (percentage) | | | | | | |
| --- | --- | --- | --- | --- | --- | --- |
|  | **Group I**  **Weight loss**  **(>5 kg)** | **Group II**  **Weight loss**  **(3-5 kg)** | **Group III**  **Weight loss**  **(1-3 kg)** | **Group IV**  **Stable weight**  **(-1 to +1 kg)** | **Group V**  **Weight gain**  **(>1 kg)** | *Missing data* |
| Weight loss, % |  |  |  |  |  |  |
| Preoperative | 5.9 ± 1.8 | 3.2 ± 0.7 | 1.6 ± 0.5 | 0.0 ± 0.5 | -2.2 ± 1.3 | *0.0* |
| Postoperative 3 months | 15.7 ± 4.0 | 16.9 ± 4.1 | 17.5 ± 3.9 | 18.0 ± 3.7 | 18.8 ± 4.0 | *1.2* |
| Postoperative 6 months | 24.4 ± 5.3 | 25.0 ± 5.7 | 25.7 ± 5.2 | 25.8 ± 5.0 | 27.0 ± 5.4 | *2.5* |
| Postoperative 9 months | 28.5 ± 6.1 | 29.1 ± 6.8 | 30.1 ± 6.5 | 30.2 ± 6.1 | 31.3 ± 6.5 | *10.0* |
| Postoperative 12 months | 30.7 ± 6.8 | 31.5 ± 7.5 | 32.0 ± 7.3 | 32.2 ± 6.9 | 33.1 ± 7.2 | *5.6* |
| Postoperative 18 months | 31.6 ± 7.8 | 32.1 ± 8.6 | 32.8 ± 8.4 | 33.1 ± 8.1 | 34.6 ± 8.0 | *16.9* |
| Postoperative 24 months | 30.7 ± 8.1 | 30.8 ± 9.3 | 32.2 ± 9.0 | 32.1 ± 8.4 | 33.8 ± 8.5 | *19.0* |
| Postoperative 36 months | 29.6 ± 8.8 | 30.4 ± 9.0 | 30.6 ± 8.9 | 31.1 ± 8.8 | 33.5 ± 8.3 | *30.5* |
| Total 36 months | 33.7 ± 8.2 | 32.6 ± 8.6 | 31.7 ± 8.7 | 31.1 ± 8.8 | 32.1 ± 8.5 | *30.5* |
| Δ BMI, kg/m^2^ |  |  |  |  |  |  |
| Preoperative | -2.6 ± 0.9 | -1.4 ± 0.2 | -0.7 ± 0.2 | 0.0 ± 0.2 | 0.9 ± 0.6 | *0.0* |
| Postoperative 3 months | -9.2 ± 1.9 | -8.5 ± 1.9 | -8.1 ± 1.8 | -7.8 ± 1.7 | -7.5 ± 1.9 | *1.2* |
| Postoperative 6 months | -12.8 ± 2.7 | -12.0 ± 2.8 | -11.6 ± 2.5 | -11.2 ± 2.5 | -11.1 ± 2.5 | *2.5* |
| Postoperative 9 months | -14.6 ± 3.3 | -13.8 ± 3.4 | -13.5 ± 3.2 | -13.1 ± 3.1 | -13.2 ± 3.2 | *10.0* |
| Postoperative 12 months | -15.5 ± 3.8 | -14.8 ± 3.9 | -14.3 ± 3.7 | -14.0 ± 3.5 | -14.0 ± 3.6 | *5.6* |
| Postoperative 18 months | -15.9 ± 4.5 | -15.0 ± 4.3 | -14.7 ± 4.3 | -14.4 ± 4.1 | -14.8 ± 4.3 | *16.9* |
| Postoperative 24 months | -15.5 ± 4.6 | -14.4 ± 4.6 | -14.4 ± 4.5 | -14.0 ± 4.1 | -14.5 ± 4.5 | *19.0* |
| Postoperative 36 months | -15.1 ± 4.9 | -14.3 ± 4.6 | -13.8 ± 4.4 | -13.4 ± 4.2 | -14.3 ± 4.4 | *30.5* |
